# Nutrient Intake, Diet Quality, and Weight Measures in Breakfast Patterns Consumed by Children Compared with Breakfast Skippers: NHANES 2001–2008

**DOI:** 10.3934/publichealth.2015.3.441

**Published:** 2015-08-03

**Authors:** Carol E. O'Neil, Theresa A. Nicklas, Victor L. Fulgoni

**Affiliations:** 1 School of Nutrition and Food Sciences, 261 Knapp Hall, Louisiana State University Agricultural Center, Baton Rouge, LA 70803, USA; 2 USDA/ARS Children's Nutrition Research Center, Department of Pediatrics, 1100 Bates Street; Baylor College of Medicine, Houston, TX 77030, USA; 3 Nutrition Impact, LLC, Battle Creek, MI 49014, USA

**Keywords:** children, adolescents, breakfast, breakfast patterns, nutrient intake, diet quality, healthy eating index, weight, cluster analysis, NHANES

## Abstract

Most studies showing that children consuming breakfast have better nutrient intakes, diet quality, and lower weight than breakfast skippers have the incorrect premise that breakfast meals are homogeneous.

The purpose of this study was to classify breakfast meals into patterns and determine the association of the breakfast patterns with daily and breakfast nutrient intakes, diet quality, and weight. Data from children (2–18 years of age; N = 14,200) participating in the National Health and Nutrition Examination Survey 2001–2008 were used. Intake was determined from one day 24-hour dietary recalls. Diet quality was measured using the Healthy Eating Index-2005 (HEI-2005). Body mass index (BMI) z-scores were determined. Twelve patterns (including No Breakfast [∼19% of population]), explaining 63% of the variance in energy from breakfast, were examined. Covariate adjusted general linear models were used to compare outcome variables of consumers of different patterns with breakfast skippers. The p value was Bonferroni corrected (< 0.05/12 = < 0.0042). Consumers of the Eggs/Grain/Meat, Poultry, Fish (MPF)/ Fruit Juice (FJ) and MPF/ Grain/FJ patterns showed higher daily intakes of saturated fats, solid fats, and sodium and lower daily intakes of added sugars than breakfast skippers. Consumers of most breakfast patterns showed higher daily intakes of some nutrients of public health concern (dietary fiber, vitamin D, calcium, and potassium); however, those consuming the Grain or MPF/Grain/FJ pattern did not. Consumers of the Grain/Lower Fat Milk (LFM)/Sweets/FJ, Presweetened (PS) Ready-to-eat Cereal (RTEC)/ LFM, RTEC/LFM, Cooked Cereal/Milk/FJ, and Whole Fruit patterns had higher total HEI-2005 scores than breakfast skippers; those consuming the MPF/ Grain/FJ pattern had lower diet quality than breakfast skippers. Consumption of the Grain/ LFM/Sweets/FJ, PSRTEC/whole milk, Soft Drinks/ FJ/Grain/Potatoes, RTEC/whole milk, and Cooked Cereal/ Milk/ FJ patterns was associated with lower BMI z-scores than seen in breakfast skippers. There are dietary and weight advantages of consuming breakfast, especially breakfasts that include grains, cereals, LFM, and fruit/ FJ, in contrast to the potential adverse effects of skipping breakfast.

## 1. Introduction

Breakfast has traditionally been considered to be the most important meal of the day [1],[2]. For children, previous studies have shown that breakfast consumption improves daily nutrient intake [1]–[5]. Breakfast consumption has been associated with lower body mass index (BMI) [1],[3]–[11] and higher levels of physical activity [8],[12], as well as improved motor skills [9], attention [13], and cognition [14]; however, these results are inconsistent and additional confirmative studies are needed. Despite these potential benefits, children often skip the breakfast meal. Breakfast skipping in children is age dependent. Data from the most recent What We Eat in America (WWEIA) component of the National Health and Nutrition Examination Survey (NHANES) data [15] showed that 2%, 11%, and 24% of males 2–5, 6–11, and 12–18 years of age (years), respectively, skipped breakfast; whereas, 5%, 9%, and 29% of females 2–5, 6–11, and 12-18 years, respectively, skipped breakfast. In children of all ages, the prevalence of skipping breakfast has increased as they grow older [3],[16]. This is especially important, not only for the reasons stated above, but since consumption of breakfast has been shown to track into young adulthood [17].

Breakfast consumers have higher intakes of many nutrients, especially micronutrients [3]–[6],[18],[19], when compared with non-consumers. An early study showed that a higher proportion of children skipping breakfast failed to meet two-thirds of the Recommended Dietary Allowance for vitamins and minerals as compared to breakfast consumers [20]. Dubois, et al. [21], however, showed that Canadian preschool children who skipped breakfast had the same energy, carbohydrate, and fat intake as those children consuming breakfast; however, protein intake was lower in breakfast skippers. Micronutrient intake was not examined in that study. Thus, the association between breakfast consumption and nutrient intake clearly depends on the type of foods consumed and what is consumed during the remainder of the day. When ready-to-eat cereals (RTEC) were consumed as part of the breakfast meal, there was a difference in nutrient intake compared with breakfast skippers or those consuming “other breakfasts” [3]. In addition to better micronutrient intakes, consumption of RTEC at breakfast has been associated with lower daily intakes of fat, saturated fatty acids (SFA), and cholesterol [3] compared to consumption of “other breakfast meals” or skipping breakfast. A higher consumption of milk has also been associated with an RTEC breakfast [22], suggesting that breakfast consumption is associated with consumption of nutrient-dense foods. A limitation of most of those studies is that breakfast and, especially, “other breakfast types” were generally not well defined by the authors and the assumption may have been made that they were homogeneous enough to include in a single group for statistical comparison.

In some studies, breakfast consumption has also been associated with lower body mass indices and other measures of adiposity in children [1],[3],[4],[6]–[11]. Ready-to-eat cereals [3],[23]–[26], including pre-sweetened RTEC (PSRTEC) [27]–[29], breakfasts have specifically been associated with lower measures of weight and adiposity. One study [3] also compared “other breakfasts” with breakfast skipping and RTEC breakfasts with weight/ adiposity parameters and showed that breakfast skippers had higher BMI z-scores and waist circumferences than those consuming RTEC or “other breakfasts.”

A recent study in adults has shown that individuals consume multiple “breakfast types” or patterns, which provide variable nutrient contributions to the overall diet and to diet quality, and have different associations with weight [30]. That study showed adults consuming containing Grain/fruit juice (FJ); PSRTEC/lower fat milk (LFM); RTEC/LFM/ Fruit/ FJ; or Cooked Cereal had lower BMIs and Waist Circumferences than breakfast skippers, but that no other patterns were associated with different measures of weight or adiposity. It is presumed that children would also show specific breakfast meal patterns with varying effects on nutrient intake, diet quality, and weight, although this has not been studied. The purposes of this study were to identify breakfast patterns consumed by a nationally representative sample of children, to examine nutrient intake resulting from consumption of breakfast patterns, and to determine the association of the breakfast patterns to total daily nutrient intake and diet quality of consumers of specific patterns vs breakfast skippers (No Breakfast pattern). Weight and adiposity measures of those consuming different breakfast patterns were also compared with breakfast skippers.

## 2. Materials and Method

### 2.1. Study Population

An overview of the National Health and Nutrition Examination Survey (NHANES), including the purpose, study population, sampling strategy, interview and physical examination procedures and response rates have been published on line [31]–[34]. In this study, data from children two to 18 years participating in the NHANES 2001–2008 were concatenated to increase sample size [35]. Those with unreliable dietary records, as judged by National Center for Health Statistics staff (n = 359), pregnant or lactating females (n = 117), and children who were breastfeeding (n = 13) were excluded from the study. The final analytic sample consisted of 14,200 children. Stringent protocols and procedures enforced by NHANES ensure confidentiality and protect individual participants [36]. As this was a secondary data analysis which lacked personal identifiers, this study did not require further institutional review [37].

### 2.2. Demographics and Dietary Information

Demographic information was determined from the NHANES interview [38]. Intake data were obtained from in-person 24-hour dietary recall interviews using an automated multiple-pass method [39],[40]. Parents/guardians of children two to five years provided the 24-hour dietary recalls; children (6 to 11 years) were assisted by an adult; and older children provided their own recall. In 2001–2002, a single 24-hour dietary recall was collected in person; however, beginning in 2003–2004, a second day of intake data was collected over the telephone. To ensure consistency, only the data from the in-person interview (first recall) were used in this study. Detailed descriptions of the dietary interview methods are provided in the NHANES Dietary Interviewers Procedure Manual, which includes pictures of the Computer-Assisted Dietary Interview system screens, measurement guides, and charts that were used to collect dietary information [41].

### 2.3. Breakfast Consumption, Food Groupings, Nutrient Analysis, Healthy Eating Index and Anthropometry

Breakfast consumption was self-reported and included consumption of any food/beverage (other than water) reported by the study participant/proxy as breakfast or brunch. The United States Department of Agriculture Food and Nutrient Database for Dietary Studies (FNDDS) foods [42] were combined into 20 breakfast food groupings [30]. All food codes fit into only one of the food groups. Ready-to-eat cereal was defined as pre-sweetened (PSRTEC) if the reference amount customarily consumed was six grams of sugar or more [43]; if there were fewer than six grams of sugar, it was defined as RTEC. Fruit juice was defined as 100% FJ [44]. Added sugars were defined by United States Department of Agriculture as all caloric sweeteners that were eaten separately or used as ingredients in processed or prepared foods [45]. Lower fat milk was defined as any milk other than whole milk. Food group intakes were determined using My Pyramid Equivalents Database (MPED) versions 1.0 [46] and 2.0 [47]; when necessary, intakes for NHANES 2005–2008 were hand matched to the same or similar foods since these data were released without an update to the MPED.

Energy and nutrient intakes were calculated using the FNDDS versions 1.0–4.1[42], for NHANES 2001–2002, 2003–2004, 2005–2006, and 2007–2008, respectively. The Vitamin D Addendum to USDA FNDDS 3.0 [42] was used to determine vitamin D content of foods and was used to hand match similar foods to determine vitamin D content of foods in previous FNDDS releases. The nutrients studied reflect the nutrients to limit (added sugars, SFA, solid fats, cholesterol, and sodium), nutrients of public health concern (dietary fiber, vitamin D, calcium and potassium), and nutrients under-consumed by some groups (vitamins A and C, folate, iron, and magnesium) [48].

Diet quality was determined using the HEI-2005 [49]. The SAS code used to calculate HEI-2005 scores was downloaded from the Center for Nutrition Policy and Promotion website [50]. It should be noted that the HEI-2005 was used, rather than the more recently released HEI-2010, since the 2005 Dietary Guidelines for Americans (DGA) were in effect during the time much of the population was participated in the NHANES. The total HEI-2005 maximum score of 100 is a composite of 12 components scores. A higher score correlates with higher compliance to the DGA and a higher diet quality. Nine of the components: total and whole fruit, total vegetables, dark green and orange vegetables and legumes, total and whole grains, milk, meat and beans, and oils address nutrient adequacy. The remaining three components: SFA; sodium; and discretionary calories (solid fats, alcohol, and added sugars [So FAAS]) are those dietary components which should be consumed in limited amounts. For the last three components, a reverse scoring is applied; thus, levels of intakes at the standard get the maximum score, with scores decreasing as intakes increases.

Height and weight were measured according to NHANES protocols [51]. Body Mass Index was calculated (wt [kg]/ht [m^2^]) and the z-score for BMI-for-age was determined using the SAS program for Growth Charts available from the Centers for Disease Control and Prevention [52]. Overweight was defined as a gender-and age-specific BMI between 85^th^ and < 95^th^ percentile and obese was defined as a BMI ≥ 95^th^ percentile [53].

### 2.4. Statistical Analyses

Breakfast consumption patterns were identified using SAS 9.2 (SAS Institute, Cary, NC; 2009) PROC CLUSTER. For each participant the percent of energy at breakfast from each of the food groupings [30] was determined. The patterns were identified by percent energy within each food grouping consumed at breakfast at the centroid of each cluster. Initially, eight, 12, and 16 patterns were evaluated; however, for subsequent analyses, the 12 pattern output (which included No Breakfast) was used since this set of breakfast patterns allowed delineation of type of RTEC and the use of LFM. Further, the 12 pattern output explained moderately more variance in energy from the breakfast meal (63% of the variance) than eight patterns (53% of the variance), but not substantially more than 16 patterns (70%). Based on their dietary intake, participants were placed into one breakfast pattern and dietary day 1 weights were used for all analyses [35].

Least-square means ±SE were calculated using PROC REGRESS of SUDAAN for dietary intake and diet quality (HEI-2005) for participants consuming each breakfast pattern. After confirming a significant overall F test for breakfast patterns (entered as categorical variables [patterns 1–12], differences for variables of interest were determined via t-test compared to the No Breakfast pattern [referred to as breakfast skippers]). Covariates included: self-reported age, gender, race/ethnicity, and the poverty income ratio (PIR), grouped into three categories (< 1.25, 1.25–3.49, and > 3.49) also served as a covariate. These values are related to the federally set poverty lines, so a PIR of < 1.25 equates to below 125% of the poverty line. Higher values mean the individuals had higher incomes. Physical activity, another covariate, was determined using a questionnaire [54]. Finally, self-reported energy intake for nutrient related variables (not for energy intake itself) was used as a covariate. The HEI-2005 was not controlled for energy intake, since the calculation of the index score controls for energy [49]. Logistic regression was used to assess the likelihood of being overweight, obese, overweight or obese for consumers of each breakfast pattern compared with breakfast skippers. For all analyses, a probability of p < 0.05 was considered significant; however, a Bonferroni correction was applied for multiple comparisons (p < 0.05/12), so the effective p value was p < 0.0042.

## 3. Results

### 3.1. Breakfast Clusters and Population Demographics

The 12 breakfast patterns identified were: 1) Grain/LFM/ Sweets/FJ (n = 2,797; 21.8% of the population); 2) No Breakfast (n = 3,018; 18.7%); 3) PSRTEC/LFM (n = 1,684; 15.2%); 4) (Grain n = 1,090; 8.2%; 5) Eggs/Grain/Meat/Poultry/Fish [MPF]/FJ (n=1,068; 6.7%); 6) PSRTEC/Whole Milk (n = 1,145; 6.5%); 7) RTEC/LFM (n = 732; 6.1%); 8) Soft Drink/FJ/Grain/Potatoes (n = 889; 5.9); 9) RTEC/Whole Milk (n = 714; 4.1%); 10) MPF/Grain/FJ (n = 617; 3.5%); 11) Cooked Cereal/Milk/FJ (n = 354; 2.5%); and 12) Whole Fruit (n = 112; 0.8%) (Table 1). The patterns varied widely by foods consumed and energy contribution of individual foods (Supplemental Tables 1 and 2, respectively). Demographics of the population (N = 14,220) by breakfast cluster are shown in Table 2

**Table 1.** The percent of breakfast calories from the 20 food groups at the centroid of the pattern, the name and assigned number of the 12 breakfast clusters, along with the number and percent of the population consuming those breakfast clusters.

Percent of Breakfast Energy at Centroid of Pattern																							
# and Name		N (%)	Milk Whole	LFM	Yogurt	Milk Products	Cheeses		MPF	Eggs	Veg & Legumes	Potatoes	Grain^1^	Cooked Cereal	RTEC	PSRTEC	Whole Fruit	FJ	Fats & Oils	Sugars & Sweets	Coffee & Tea	Soft/Fruit Drinks	Other Drinks
1	Grain/Low Fat Milk/Sweets/Fruit Juice	2,797 (21.81)	4	13	1	0	2		3	2	1	1	47	0	1	3	2	7	3	10	0	1	0
2	No Breakfast	3,018 (18.65)																					
3	PSRTEC/Low Fat Milk	1,684 (15.17)	0	34	0	0	0	0		0	0	0	1	0	0	58	1	4	0	0	0	0	0
4	Grain	1,090 (8.20)	0	2	0	0	0	0		0	0	0	88	0	0	0	0	0	2	1	1	4	0
5	Eggs/Grain/MPF/FJ	1,068 (6.67)	5	8	0	0	1	7		48	1	0	15	0	1	1	1	6	2	2	0	2	0
6	PSRTEC/Whole Milk	1,145 (6.53)	39	0	0	0	0	0		0	0	0	0	0	0	55	1	3	0	0	0	1	0
7	RTEC/Low Fat Milk	732 (6.10)	0	52	1	0	0	0		0	1	0	3	0	31	0	4	5	0	2	0	0	0
8	Soft Drink/FJ/Grain/Potatoes	889 (5.87)	1	4	8	5	1	4		4	5	7	8	0	1	6	3	14	1	2	3	23	0
9	RTEC/Whole Milk	714 (4.14)	53	0	0	0	0	0		0	0	0	6	0	22	6	4	3	1	2	1	1	0
10	MPF/Grain/FJ	617 (3.54)	3	3	0	0	1	55		3	1	4	14	0	1	1	0	6	1	2	1	3	0
11	Cooked Cereal/Milk/FJ	354 (2.52)	5	9	0	0	0	0		1	0	0	4	65	0	1	2	7	1	4	0	1	0
12	Whole Fruit	112 (0.79)	0	0	0	1	1	0		0	0	0	0	0	0	0	96	0	0	2	0	0	0
All		14,220 (100.00)	8	15	1	0	1	4		5	1	1	25	2	4	17	3	6	1	4	0	3	0

Source: Children two to eighteen years of age participating in NHANES, 2001-2008, excludes pregnant/lactating females and breast feeding children.

^1^Grain not cereal

Abbreviations: LFM = lower fat milk; MPF = meat, poultry, fish; veg = vegetable; RTEC = ready-to-eat cereal; PSRTEC = presweetened ready-to-eat cereal; FJ = fruit juice

**Table 2.** Demographics of children 2–18 years of age participating in NHANES 2001-2008 by assigned breakfast cluster.

Variable	Cluster											
	Grain/lower fat milk/sweets/FJ	No breakfast	PSRTEC/lower fat milk	Grain	Eggs/Grain/mpf/FJ	PSRTEC/whole milk	RTEC/lower fat milk	Soft drink/FJ /grain/potatoes	RTEC/whole milk	Mpf/grain/FJ	Cooked cereal/milk/FJ	Whole fruit
n	2797	3018	1684	1090	1068	1145	732	889	714	617	354	112
Male (%)	50.3 ± 1.1	50.4 ± 1.5	55.9 ± 1.7	44.5 ± 2.2	50.7 ± 2.3	53.7 ± 2.5	46.9 ± 2.5	48.7 ± 2.6	52.2 ± 3.1	54.0 ± 3.4	51.0 ± 3.6	41.9 ± 8.4
Female (%)	49.7 ± 1.1	49.6 ± 1.5	44.1 ± 1.7	55.5 ± 2.2	49.3 ± 2.3	46.3 ± 2.5	53.1 ± 2.5	51.3 ± 2.6	47.8 ± 3.1	46.0 ± 3.4	49.0 ± 3.6	58.1 ± 8.4
White (%)	64.4 ± 1.9	54.3 ± 2.7	72.8 ± 2.1	69.2 ± 2.7	56.5 ± 3.4	45.4 ± 3.0	68.4 ± 2.9	62.8 ± 2.8	43.7 ± 3.3	50.6 ± 4.3	62.6 ± 3.9	60.2 ± 7.6
Black (%)	12.2 ± 1.2	19.1 ± 1.6	9.7 ± 1.2	10.9 ± 1.4	15.3 ± 1.8	26.5 ± 2.4	6.2 ± 1.1	13.7 ± 1.8	17.4 ± 2.1	23.5 ± 3.1	18.4 ± 2.8	8.3 ± 2.3
Hispanic (%)	16.5 ± 1.4	20.1 ± 2.1	12.6 ± 1.4	14.0 ± 1.3	21.6 ± 2.5	21.6 ± 2.3	18.4 ± 2.0	19.2 ± 2.0	30.1 ± 2.8	18.8 ± 2.6	13.8 ± 2.2	21.5 ± 5.9
Other (%)	6.9 ± 0.9	6.4 ± 1.1	5.0 ± 0.9	6.0 ± 1.2	6.6 ± 1.5	6.5 ± 1.2	7.0 ± 1.7	4.3 ± 1.0	8.7 ± 2.0	7.2 ± 1.8	5.3 ± 1.4	9.9 ± 4.2
PIR	2.8 ± 0.1	2.3 ± 0.1	2.7 ± 0.1	2.8 ± 0.1	2.4 ± 0.1	1.8 ± 0.1	2.7 ± 0.1	2.6 ± 0.1	1.8 ± 0.1	2.2 ± 0.1	2.5 ± 0.2	3.0 ± 0.2
Sedentary (%)	13.7 ± 0.9	14.4 ± 0.9	12.7 ± 1.5	15.2 ± 1.7	15.0 ± 1.9	15.5 ± 2.0	12.2 ± 1.6	9.9 ± 1.2	19.6 ± 2.4	11.8 ± 2.3	20.5 ± 4.2	11.6 ± 4.2
Light (%)	21.4 ± 1.1	19.8 ± 1.3	23.3 ± 1.8	19.9 ± 1.9	19.6 ± 2.1	19.7 ± 2.5	16.8 ± 2.0	15.9 ± 2.0	18.4 ± 2.4	18.2 ± 3.0	20.1 ± 3.2	8.4 ± 3.1
Med-Vig (%)	64.9 ± 1.3	65.8 ± 1.4	64.0 ± 2.0	64.9 ± 2.4	65.4 ± 2.5	64.8 ± 2.0	71.0 ± 2.7	74.2 ± 2.3	62.0 ± 2.9	70.1 ± 3.8	59.5 ± 4.5	80.0 ± 5.5
Alcohol No (%)	95.3 ± 0.8	95.0 ± 0.7	97.2 ± 0.6	98.1 ± 0.4	96.4 ± 0.9	96.1 ± 1.2	98.3 ± 0.7	96.8 ± 0.8	97.4 ± 0.9	96.8 ± 0.6	96.8 ± 1.7	90.8 ± 4.4
Alcohol Yes (%)	4.7 ± 0.8	5.1 ± 0.7	2.8 ± 0.6	1.9 ± 0.4	3.6 ± 0.9	3.9 ± 1.2	1.7 ± 0.7	3.2 ± 0.8	2.6 ± 0.9	3.2 ± 0.6	3.2 ± 1.7	9.2 ± 4.4
% Energy FF	9.8 ± 0.9	17.3 ± 1.1	10.5 ± 1.0	12.9 ± 1.1	14.4 ± 1.3	11.7 ± 1.2	9.8 ± 1.2	16.2 ± 1.1	9.1 ± 1.2	17.2 ± 2.1	8.0 ± 1.3	15.6 ± 4.1

Data Source: Children two to eighteen years of age participating in NHANES, 2001-2008, excludes pregnant/lactating females and breast feeding children.

Abbreviations: FJ = Fruit Juice; PSRTEC = presweetened ready-to-eat cereal; mpf = meat, poultry, fish; RTEC = ready-to-eat cereal; n = Number; PIR = Poverty Index Ratio; Vig = Vigorous; FF= Fast Food; HEI = Healthy Eating Index

**Table 3.** Consumption of Energy, Protein, and Nutrients to Limit Breakfast Pattern^1^ Consumed by Children 2–18 Years of Age Participating in the 2001–2008 National Health and Nutrition Examination Survey.

Total Daily Consumption								
#	ClusterName	Energy(kcal)	Protein(g)	AddedSugars (tsp)	SFA (g)	Solid Fat (g)	Cholesterol(mg)	Sodium (mg)
		Mean±SE	Mean±SE	Mean±SE	Mean±SE	Mean±SE	Mean±SE	Mean±SE
Breakfast Consumption Only^1^								
1	Gr/lower fat milk/sweets/FJ	483±7	14.0±0.3	4.7±0.2	5.4±0.2	11.1±0.3	56±3	639±14
2	No breakfast							
3	PSRTEC/lower fat milk	341±8	11.6±0.3	4.4±0.1	3.1±0.1	4.9±0.2	25±4	409±12
4	Gr	389±11	8.5±0.4	4.7±0.2	4.2±0.2	11.7±0.5	26±2	519±22
5	Eggs/Grain/MPF/FJ	509±17	24.8±0.7	1.9±0.2	9.0±0.3	16.4±0.6	421±11	909±27
6	PSRTEC/whole milk	341±8	10.2±0.3	4.5±0.1	4.3±0.1	7.1±0.2	22±1	339±8
7	RTEC/lower fat milk	318±11	12.9±0.4	2.8±0.2	3.8±0.2	5.9±0.4	27±1	405±18
8	Soft drink/FJ/Gr/potatoes	359±16	9.2±0.5	5.9±0.4	3.6±0.2	6.7±0.5	57±7	415±24
9	RTEC/whole milk	340±10	12.3±0.4	2.2±0.1	5.7±0.2	9.7±0.4	36±2	381±15
10	MPF/Gr/FJ	571±22	24.4±1.0	2.8±0.2	9.5±0.4	20.1±0.8	108±10	1116±33
11	Cooked Cereal/Milk/FJ	384±14	12.5±0.6	3.4±0.3	3.4±0.2	5.0±0.4	25±3	401±18
12	Whole Fruit	97±12	1.2±0.4	0.7±0.2	0.8±0.2	1.6±0.4	7±3	68±18

#	ClusterName	Energy(kcal)	Protein(g)	AddedSugars (tsp)	SFA (g)	Solid Fat (g)	Cholesterol(mg)	Sodium (mg)
		Mean±SE	Mean±SE	Mean±SE	Mean±SE	Mean±SE	Mean±SE	Mean±SE
Total Daily Consumption^1^								
1	Gr/LMF/sweets/FJ	2152±22^*^	73.2±1.0^*^	20.9±0.4^*^	25.8±0.2	46.1±0.6	205±4	3106±27
2	No breakfast	1762±28	59.7±1.3	23.5±0.5	26.7±0.4	47.2±0.8	199±4	3087±34
3	PSRTEC/LFM	2060±30^*^	72.2±1.2^*^	23.0±0.5	24.9±0.3^*^	42.2±0.7^*^	185±5	3032±49
4	Gr	2040±35^*^	64.7±1.5	22.0±0.5	26.2±0.4	50.4±0.8	187±7	3098±42
5	Eggs/Gr/MPF/FJ	2183±63^*^	83.7±2.3^*^	18.4±0.9^*^	29.3±0.5^*^	51.5±0.9^*^	574±14^*^	3244±46^*^
6	PSRTEC/whole milk	2145±36^*^	72.6±1.3^*^	22.9±0.5	26.8±0.3	47.4±0.8	189±4	3003±49
7	RTEC/LFM	1903±37^*^	69.1±1.4^*^	20.6±0.7	25.7±0.4	44.1±0.9	191±5	3021±47
8	Soft Drink/FJ/Gr/potatoes	2014±47^*^	67.2±2.0^*^	24.5±0.6	25.7±0.4	44.8±0.8	227±7^*^	3045±52
9	RTEC/whole milk	1987±41^*^	70.7±1.9^*^	19.5±0.5^*^	28.6±0.4^*^	49.6±1.0	217±7	3092±89
10	MPF/Gr/FJ	2269±95^*^	85.3±3.8^*^	18.8±1.0^*^	29.3±0.6^*^	56.1±1.4^*^	252±9^*^	3452±78^*^
11	Cooked cereal/milk/FJ	2020±56^*^	70.0±2.4^*^	20.6±1.1	24.3±0.7	42.2±1.4^*^	186±10	2962±67
12	Whole fruit	1630±75	59.5±3.9	19.3±1.5	27.1±1.1	48.3±2.6	203±11	3063±77

Abbreviations: kcals = kilocalories, tsp = teaspoon, SFA = saturated fatty acids; g= grams; mg = milligrams; SE = standard error; FJ = fruit juice; PSRTEC = presweetened ready-to-eat cereal; MPF = meat, poultry, fish; RTEC = ready-to-eat cereal

^1^Covariates: Age, gender, race/ethnicity, poverty income ratio grouped into three categories as (< 1.25, 1.25–3.49, and >3.49), current smoking status (yes/no) (adults only), physical activity level (sedentary, moderate and vigorous), alcohol intake (g/d), energy intake for nutrient related variables

^*^Statistically different from No Breakfast; with the Bonferroni correction effective p < 0.0042; comparison for total daily consumption only

**Table 4.** Consumption of Nutrients of Public Health Concern and Nutrients of Potential Concern to Some Groups^1^ by Breakfast Cluster by Children participating in the 2001–2008 National Health and Nutrition Examination Survey.

		Nutrients of Public Health Concern defined by the 2010 Dietary Guidelines for Americans				Shortfall Nutrients Identified by the 2010 Dietary Guidelines for Americans				
#	Cluster Name	Dietary Fiber (g)	Vitamin D^2^ (mcg)	Calcium (mg)	Potassium (mg)	Vitamin A (RAE mcg)	Vitamin C(mg)	Folate DFE (mcg)	Iron (mg)	Magnesium (mg)
		Mean±SE	Mean±SE	Mean±SE	Mean±SE	Mean±SE	Mean±SE	Mean±SE	Mean±SE	Mean±SE
Breakfast Consumption Only^1^										
1	Gr/LFM/sweets/FJ	2.6±0.1	2.23±0.06	317.1±6.5	535.4±9.2	217.9±5.3	24.4±1.3	156.6±4.7	4.2±0.1	52.7±1.2
2	No breakfast									
3	PSRTEC/LFM	2.6±0.1	4.1±0.1	380.3±10.9	533.9±13.5	353.0±10.4	19.6±1.0	428.0±17.1	8.5±0.2	57.2±1.7
4	Gr	2.1±0.1	0.4±0.04	126.9±8.9	210.5±9.8	126.8±9.0	7.0±1.0	100.6±4.1	3.1±0.1	28.3±1.1
5	Eggs/Gr/MPF/FJ	1.5±0.1	3.2±0.2	314.9±13.7	603.6±19.8	260.8±7.8	25.9±2.0	112.4±4.8	3.8±0.1	51.6±1.5
6	PSRTEC/whole milk	2.1±0.1	4.1±0.1	314.0±7.8	445.1±11.0	286.7±8.8	20.0±1.6	430.9±22.6	8.3±0.4	48.1±1.8
7	RTEC/LFM	2.6±0.2	4.2±0.1	411.8±13.9	644.6±23.9	308.8±10.4	23.7±2.7	287.6±22.0	6.7±0.4	63.2±2.3
8	SD/FJ/Gr/potatoes	2±0.1	1.2±0.1	192.1±10.5	502.2±23.5	125.3±9.1	41.1±2.5	99.5±8.0	2.6±0.2	44.0±2.5
9	RTEC/whole milk	2.1±0.2	4.4±0.2	386.9±14.4	569.3±18.3	242.6±8.7	15.1±1.2	283.5±13.2	6.9±0.3	52.6±1.8
10	MPF/Gr/FJ	1.9±0.2	1.6±0.2	204.8±13.9	587.9±29.4	97.3±11.3	24.0±3.0	103.8±9.5	3.5±0.2	47.7±1.9
11	Cooked cereal/milk/FJ	4.6±0.2	1.7±0.2	349.7±18.7	561.3±30.6	406.3±30.5	26.1±4.6	195.5±11.7	7.2±0.4	84.8±3.4
12	Whole fruit	3.5±0.2	0.3±0.1	52.2±10.9	371.7±38.2	57.0±21.5†	36.3±7.0	36.2±5.9	0.7±0.1	24.6±2.3

		Nutrients of Public Health Concern defined by the 2010Dietary Guidelines for Americans				Shortfall Nutrients Identified by the 2010 Dietary Guidelines for Americans				
#	Cluster Name	Dietary Fiber (g)	Vitamin D (mcg)	Calcium (mg)	Potassium (mg)	Vitamin A (RAE mcg)	Vitamin C (mg)	Folate DFE (mcg)	Iron (mg)	Magnesium (mg)
		Mean±SE	Mean±SE	Mean±SE	Mean±SE	Mean±SE	Mean±SE	Mean±SE	Mean±SE	Mean±SE
Total Daily Consumption^1^										
1	Gr/LFM/sweets/FJ	12.8±0.2^*^	5.7±0.1^*^	1030.8±14.6^*^	2237.6±27.3^*^	581.4±11.3^*^	82.1±2.3	491.8±7.4^*^	13.8±0.2^*^	227.8±2.4^*^
2	No breakfast	12.1±0.2	3.7±0.2	858.4±20.4	2061.9±27.8	427.9±17.1	80.0±3.5	404.9±10.2	12.0±0.2	211.3±2.6
3	PSRTEC/LFM	13.7±0.3^*^	7.7±0.2^*^	1143.0±19.9^*^	2366.3±31.8^*^	746.6±15.5^*^	85.4±2.4	785.0±18.0^*^	18.7±0.2^*^	247.6±3.4^*^
4	Gr	12.0±0.2	3.3±0.2	824.8±20.7	1870.4±30.5^*^	492.1±22.0	71.0±4.0	439.7±10.7	12.7±0.2	201.5±4.1
5	Eggs/Gr/MPF/FJ	11.4±0.3	6.4±0.3^*^	974.9±26.2^*^	2282.0±45.1^*^	599.3±18.4^*^	85.2±3.8	406.1±12.0	13.0±0.3	219.1±3.8
6	PSRTEC/whole milk	12.7±0.3	7.4±0.3^*^	1018.7±21.7^*^	2221.3±37.5^*^	615.1±21.0^*^	86.6±4.0	769.4±20.4^*^	18.2±0.4^*^	231.7±3.6^*^
7	RTEC/LFM	13.7±0.3^*^	7.9±0.3^*^	1200.0±28.5^*^	2540.1±51.6^*^	739.4±26.5^*^	90.6±4.0	652.0±27.2^*^	17.0±0.5^*^	253.1±4.0^*^
8	SD/FJ/Gr/potatoes	11.9±0.2	4.5±0.2	957.7±23.9^*^	2208.1±46.4^*^	528.8±26.5^*^	108.7±5.0^*^	439.4±13.3	12.5±0.2	222.5±3.9
9	RTEC/whole milk	12.5±0.3	8.1±0.2^*^	1150.8±17.9^*^	2454.7±58.9^*^	638.9±46.0^*^	88.8±4.9	644.6±17.7^*^	17.0±0.4^*^	241.7±2.8^*^
10	MPF/Gr/FJ	11.4±0.3	4.6±0.3	812.9±31.7	2163.7±49.6	395.4±22.3	72.9±4.0	398.8±20.8	12.9±0.6	208.4±3.0
11	Cooked cereal/milk/FJ	15.8±0.6^*^	5.6±0.5^*^	1115.8±43.4^*^	2403.7±71.9^*^	843.4±41.5^*^	90.6±7.4	524.6±16.4^*^	17.6±0.6^*^	276.4±7.3^*^
12	Whole fruit	15.2±0.5^*^	3.5±0.4	856.6±45.3	2416.5±94.6^*^	450.3±48.9	97.7±14.0	442.3±43.9	12.4±0.4	230.2±6.3^*^

Nutrients of public health concern and underconsumed nutrients were identified by the 2010 Dietary Guidelines for Americans.

Abbreviations: g = grams, SE = standard error, mcg = micrograms, mg = milligrams, RAE = retinol activity equivalents, DFE = dietary folate equivalents; Gr = Grain; LFM = lower fat milk; FJ = fruit juice; PSRTEC = pre-sweetened ready-to-eat cereal; MPF = meat, poultry, fish; RTEC = ready-to-eat cereal; SD = soft drinks

^1^Covariates: Age, gender, race/ethnicity, poverty income ratio grouped into three categories as (< 1.25, 1.25–3.49, and > 3.49), physical activity level (sedentary, moderate and vigorous), alcohol intake (g/d), energy intake for nutrient related variables, ratio of reported energy intake to predicted energy intake from IOM equations (predicted energy intake is TEE for overweight children and EER otherwise).

^2^Vitamin D = D2 + D3

∗ Statistically different from No Breakfast; with the Bonferroni correction effective p < 0.0042; † not statistically different from No Breakfast

**Table 5.** Weight parameters by breakfast pattern in children 2–18 years of age participating in 2001–2008 NHANES.

	%	Pattern	Weight (kg)	BMI	BMI z-score	Overweight	Obese	Overweight/Obese
			LSM±SE	LSM±SE	LSM±SE	%±SE	%±SE	%±SE
1	21.81	Gr/LFM/Sweets/FJ	42.4±0.4^*^	19.7±0.1^*^	0.4±0.04^*^	16±1	14±1	30±2
2	18.65	No Breakfast	44.9±0.5	20.6±0.2	0.6±0.04	17±1	19±1	36±2
3	15.17	PSRTEC/LFM	43.3±0.5	19.9±0.2	0.5±0.1	16±2	16±2	32±2
4	8.20	Gr	43.7±0.8	20.1±0.2	0.5±0.1	19±2	17±2	36±3
5	6.67	Eggs/Gr/MPF/FJ	44.6±0.7	20.4±0.2	0.5±0.1	13±2	19±2	32±2
6	6.53	PSRTEC/whole milk	41.4±0.7^*^	19.3±0.3^*^	0.2±0.1^*^	10±1^*^	14±2	25±3^*^
7	6.10	RTEC/LFM	42.6±0.8	19.8±0.3	0.4±0.1	11±2^*^	15±2	26±3^*^
8	5.87	Soft Drinks/FJ/Gr/Potatoes	43.1±0.7	20.0±0.2	0.4±0.1^*^	15±2	14±2	29±2
9	4.14	RTEC/Whole Milk	41.5±0.4^*^	19.3±0.2^*^	0.2±0.1^*^	13±2	10±2^*^	23±2^*^
10	3.54	MPF/Gr/FJ	42.8±0.9	20.0±0.4	0.4±0.1	13±2	18±3^*^	30±4
11	2.52	Cooked Cereal/Milk/FJ	41.4±0.7^*^	19.4±0.2^*^	0.3±0.1^*^	16±4	8±1	23±4^*^
12	0.79	Whole Fruit	45.5±1.8	20.1±0.5	0.6±0.2	21±5	15±4	36±7

Covariates: age, gender, race/ethnicity, poverty income ratio grouped into three categories as (< 1.25, 1.25–3.49, and >3.49), current physical activity level (sedentary, moderate and vigorous), alcohol intake (g/d), and energy

^*^ = significantly different from no breakfast at p< 0.0042

Abbreviations: LSM = least square mean; SE = standard error; Gr = grain; LFM = lower fat milk; FJ = fruit juice; PSRTEC = pre-sweetened ready-to-eat cereal; MPF = meat, poultry, fish; RTEC = ready-to-eat cereal

### 3.2. Energy, Protein, Nutrient Intake, and Diet Quality

Table 3 presents the absolute intake of energy, protein, and nutrients to limit for the breakfast meal only and for the entire day. To help the reader understand the contribution of the breakfast meal to the entire day's intake, the energy and nutrients have been converted to percentages in this section. The percent energy contributed by the breakfast meal to the daily total varied widely among those consuming different breakfast patterns. The Whole Fruit breakfast pattern was associated with the lowest (6%) and MPF/Gr/FJ breakfast pattern was associated with the highest (25%) percent of daily energy intake. A Whole Fruit breakfast also contributed the lowest percent protein (2%), added sugars (3.6%), SFA (0.3%), solid fat (3%), cholesterol (3%) and sodium (2%) to the daily intake of these nutrients. The percent of protein contributed by the breakfast meal to the daily total was highest among consumers of the Eggs/Grain/MPF/FJ breakfast pattern (30%). A Soft Drink/FJ/Grain/Potatoes breakfast contributed the highest percent intake of added sugars (24%) to the daily intake. An Eggs/Grain/MPF/FJ breakfast contributed the highest (31%) percent total daily intake of SFA and cholesterol (73%). The MPF/Gr/FJ breakfast pattern contributed the highest (36%) percent total daily intake of solid fat and sodium (32%).

Table 4 presents the absolute intake of nutrients of public health concern and shortfall nutrients for the breakfast meal only and for the entire day. To help the reader understand the contribution of the breakfast meal to the entire day's intake, the energy and nutrients have been converted to percentages in this section. For the breakfast meal only, consumers of all breakfast patterns had higher intakes of all nutrients examined than breakfast skippers, except for those consuming the Whole Fruit breakfast pattern — they had a vitamin A intake which was not different from breakfast skippers. The Cooked Cereal/Milk/FJ breakfast contributed the highest (29%) and the Eggs/Grain/MPF/FJ breakfast contributed the lowest (13%) percent fiber to the daily intake of dietary fiber. Except for the Cooked Cereal/Milk/FJ pattern, only the breakfast patterns with milk (PSRTEC/LFM; PSRTEC/Whole Milk; RTEC/LFM; RTEC/Whole Milk) contributed high percentages of vitamin D to the daily intake. The Whole Fruit pattern contributed the lowest percentage (0.6%) of calcium to the total daily intake; and the Grain pattern contributed the lowest percentage of potassium (11%) to the total daily intake.

For the entire day, energy intake ranged from approximately 1630 kcals/day for those consuming the Whole Fruit breakfast pattern to 2270 kcals/day for those consuming the MPF/Grain/FJ breakfast pattern (Table 3). For those in all patterns, except those in the Whole Fruit breakfast pattern, higher daily energy intakes were seen compared to breakfast skippers. Mean daily protein intake for those consumers of all breakfast patterns, except Grains and Whole Fruit, was higher than that seen among breakfast skippers. For those in the 12 breakfast patterns, daily intake of added sugars ranged from approximately 19–24 tsp/day. For those in the Grain/LFM/Sweets/FJ, Eggs/Grain/MPF/FJ, and RTEC/Whole Milk breakfast patterns, daily intake of added sugars was lower than that seen among breakfast skippers. Only the consumers of the PSRTEC/LFM breakfast pattern had a lower daily intake of SFA than breakfast skippers; whereas, consumers of the Eggs/Grain/MPF/FJ, RTEC/Whole Milk, and MPF/Grain/FJ breakfast patterns had a higher daily intake of SFA than breakfast skippers. Only consumers of the Eggs/Grain/MPF/FJ and the MPF/Grain/FJ breakfast patterns had higher in total sodium intakes than breakfast skippers.

When total mean daily intake of dietary fiber was considered, those consuming the PSRTEC/LFM, RTEC/LFM, Cooked Cereal/Milk/FJ, and Whole Fruit breakfast patterns consumed more dietary fiber than breakfast skippers (Table 4); however, mean intake of consumers of all breakfast patterns was low. When mean daily Vitamin D intake by consumers of the breakfast patterns were compared with breakfast skippers, only those placed in the Grain, Soft Drinks/FJ/Grain/potatoes, MPF/Grain /FJ, and Whole Fruit breakfast patterns did not have higher intakes of Vitamin D intake than breakfast skippers. Consumers in all breakfast patterns except Grain, MPF/Grain/FJ, and Whole Fruit had higher mean daily intakes of calcium than breakfast skippers. Consumers in all breakfast patterns except Grain (lower than breakfast skippers) and MPF/Grain/FJ (not different from breakfast skippers) had higher intakes of potassium than breakfast skippers. Also presented in Table 4 are daily intakes of shortfall nutrients, by those in the different breakfast patterns compared with breakfast skippers. Only consumers in the Grain, MPF/Grain/FJ, and Whole Fruit breakfast patterns did not have daily intakes of vitamin A that were higher than breakfast skippers; whereas, only consumers in the Soft Drink/FJ/Grain/Potatoes breakfast pattern had a higher mean intake of vitamin C than breakfast skippers. For folate, iron, and magnesium, consumers of the Gr/LFM/sweets/FJ, PSRTEC/LFM, PSRTEC/Whole Milk, RTEC/LFM, RTEC/Whole Milk, and Cooked Cereal/Milk/FJ breakfast patterns had higher intakes than breakfast skippers. Finally, consumers of the Whole Fruit breakfast pattern also had higher intakes of magnesium, when compared with breakfast skippers.

### 3.3. Overall Diet Quality

**Figure 1.**
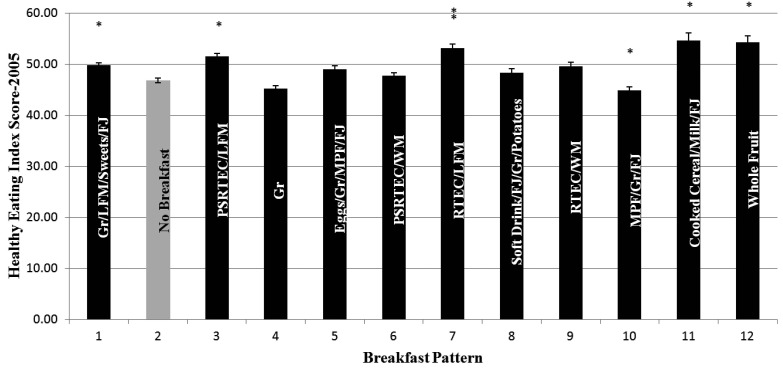
Healthy Eating Indices by Breakfast Cluster for children 2–18 years of age participating in the 2001-2008 National Health and Nutrition Examination Survey. Asterisk means that values are significantly different from No Breakfast (Pattern 2)—Bonferroni corrected p = 0.0042. Breakfast Patterns: 1 = Grain/Lower Fat Milk/Sweets/Fruit Juice (FJ); 2 = No Breakfast; 3 = Pre-sweetened ready-to-eat cereal (PSRTEC)/Lower Fat Milk; 4 = Grains; 5 = Eggs/Grain/Meat, Poultry, Fish (MPF); 6 = PSRTEC/Whole Milk; 7 = RTEC/Lower Fat Milk; 8 = Soft Drinks/FJ/Grain/Potatoes; 9 = RTEC/Whole Milk; 10 = MPF/Grain/FJ; 11 = Cooked Cereal/Milk/FJ; 12 = Whole Fruit.Covariates: Age, gender, race/ethnicity, poverty income ratio grouped into three categories as (<1.25, 1.25–3.49, and >3.49), current smoking status (yes/no) (adults only), physical activity level (sedentary, moderate and vigorous), alcohol intake (g/d). Note that energy was not used as a covariate since the HEI score itself is controlled for energy. Abbreviations: Gr= Grain; LFM = lower fat milk; FJ = Fruit Juice; PSRTEC = presweetened ready-to-eat-cereal; MPF = meat, poultry, fish; WM = whole milk; RTEC = ready-to-eat cereal. *Significantly different from no breakfast; Bonferroni corrected, p < 0.0042

Figure 1 shows the HEI-2005 by breakfast pattern. On average, HEI-2005 scores were low, with consumers of the breakfast patterns (No Breakfast, Grain, Eggs/Grain/MPF/FJ, PSRTEC/Whole Milk, Soft Drinks/FJ/Grain/Potatoes, and MPF/Grain/FJ) showing a total daily score of less than 50 (out of a maximum of 100). Those consuming the breakfast patterns: Grain/LFM/sweets/FJ, PSRTEC/LFM, RTEC/LFM, Cooked Cereal/Milk/FJ, and Whole Fruit, had higher HEI-2005 scores than breakfast skippers; whereas, those consuming the MPF/Grain/FJ breakfast pattern had a lower HEI-2005 score than breakfast skippers.

### 3.4. Weight Parameters

Table 5 shows weight (kg), BMI, BMI z-score, and the percentage of the population that were overweight, obese, or overweight or obese by breakfast pattern. Mean BMI z-scores were lower among consumers of five of the breakfast patterns, Grain/LFM/Sweets/FJ, PSRTEC/Whole Milk, Soft Drinks/FJ/Grain/Potatoes, RTEC/Whole Milk, and Cooked Cereal/Milk/FJ, when compared to breakfast skippers. The percentage of overweight or obese children was lower in consumers of four of the breakfast patterns, PSRTEC/Whole Milk, RTEC/LFM, RTEC/Whole Milk, and Cooked Cereal/Milk/FJ when compared with breakfast skippers.

**Figure 2.**
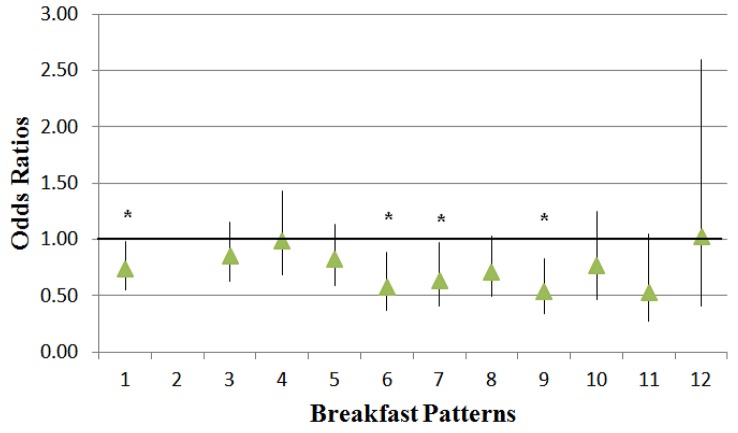
Odds ratios of breakfast patterns (compared with No Breakfast, Pattern 2) for overweight or obese children; an * indicates a significant (p < 0.0042) lower risk of overweight or obesity. Breakfast Patterns: 1 = Grain/Lower Fat Milk/Sweets/Fruit Juice (FJ); 2 = No Breakfast; 3 = Pre-sweetened ready-to-eat cereal (PSRTEC)/Lower Fat Milk; 4 = Grains; 5 = Eggs/Grain/Meat, Poultry, Fish (MPF); 6 = PSRTEC/Whole Milk; 7 = RTEC/Lower Fat Milk; 8 = Soft Drinks/FJ/Grain/Potatoes; 9 = RTEC/Whole Milk; 10 = MPF/Grain/FJ; 11 = Cooked Cereal/Milk/FJ; 12 = Whole Fruit.Data source: 2001–2008 National Health and Nutrition Examination Survey, children 2–18 years of age. Covariates: age, gender, race/ethnicity, poverty income ratio, physical activity, and alcohol intake.

Those consuming the Grain/LFM/Sweets/FJ (Odds Ratio [OR]: 0.73; 95^th^ CI [Confidence Interval]: 0.55–0.98), PSRTEC/Whole Milk (OR: 0.57; 95^th^ CI: 0.37–0.88), RTEC/LFM (OR: 0.63; 95^th^ CI: 0.41–0.98), or RTEC/Whole Milk (OR: 0.54; 95^th^ CI: 0.35–0.83) breakfast patterns were 27%, 43%, 37%, or 46%, respectively less likely to be overweight or obese than breakfast skippers (Figure 2).

## 4. Discussion

A novel contribution of this study was the identification of 12 distinct breakfast patterns and their differential association with nutrient intakes, diet quality, and weight in children. This study showed that nearly 19% of the population skipped breakfast (No Breakfast Pattern); 11 specific breakfast patterns consumed by children were also identified. Mean nutrient consumption among the consumers assigned to the different patterns varied both for the breakfast meal and for the day; not all patterns differed significantly from breakfast skippers. Diet quality also varied among the breakfast consumption patterns. Only consumers of breakfast patterns characterized by cereal or grain intake (Gr/LFM/Sweets/FJ; PSRTEC/whole milk; RTEC/whole milk; Cooked Cereal/Milk/FJ), ironically except the actual “Grains,” pattern, which included primarily sweetened grains such as doughnuts, cinnamon buns, and muffins and the RTEC with LFM pattern, had lower mean BMI values than breakfast skippers.

Breakfast consumers have been shown repeatedly to have higher daily intakes of vitamins and minerals, than non-consumers [1]–[6], [19], [20], [22], [21], [23]–[26]. The previous literature suggests that children who skip breakfast may not make up for missed nutrients during the day [20], underscoring the importance of the breakfast meal. However, our study suggested this was true for consumers of some breakfast patterns, but not all. Consumers of the majority of breakfast patterns had higher daily intakes of most nutrients of public health concern, as defined by the 2010 DGA [48], as well as some potentially underconsumed nutrients by some sub-populations [48] when compared with breakfast skippers. The varying results with different breakfast patters suggested that the composition of the breakfast meal is important. This finding was not surprising since the foods contributing the highest levels of energy in the Grain pattern were breakfast tarts, doughnuts, pancakes and waffles, pizza, and noodle soups. The foods contributing the highest levels of energy in the MPF/Grain/FJ pattern were sausage biscuits, pork sausage, whole milk, French fries, bacon, and sausage and full-fat cheese on English muffins. These foods tend to be energy dense and may contribute high levels of nutrients to limit, such as SFA and sodium.

In this study, the finding that children who consumed RTEC, including PSRTEC, breakfast patterns had high intakes of most micronutrients of concern confirmed results from other studies [3], [22], [23], [25]–[27]. Virtually all RTEC are vitamin and mineral fortified, so these results were not surprising. Further, consumption of RTEC has been shown to increase consumption of fluid milk [22], which contributes vitamins A and D, calcium, and potassium to the diet.

None of the breakfast patterns showed a mean daily intake of added sugars that exceeded the Institute of Medicine's threshold of 25% of energy [55]. This included the patterns with PSRTEC, soft drinks, and sweets suggesting a moderate intake of these foods or consumption of foods lower in added sugars throughout the day. It should be noted however, that the mean intake of added sugars ranged from 13% (MPF/Grain/FJ) to 21% (breakfast skippers). Consumers of all patterns, including the No Breakfast pattern, exceeded the total daily recommendations for percent energy from SFA [55] and for sodium [56], suggesting that even those children consuming breakfast patterns characterized by foods low in these nutrients, such as those patterns including LFM, RTEC or cooked cereal, and fruit or FJ, need to reduce intake of foods high in SFA and sodium at other meals and snacks throughout the day.

Results from this study suggested that simply consuming breakfast was not associated with a higher diet quality when compared with breakfast skippers, but that the specific foods or food groups consumed at the breakfast meal may have influenced total diet quality. It is also possible that those consuming foods generally regarded as “healthy” at the breakfast meal have an overall “healthier” eating pattern throughout the day; although overall, children had poor diet quality, regardless of the breakfast pattern consumed. Further studies are needed to confirm this. There is no standard definition of a high quality or nutrient-dense breakfast, despite the recommendation from the 2010 DGA to consume a “nutrient-dense” breakfast. The 2010 DGA provide no recommendations for consumption of specific nutrients or foods to be consumed at breakfast [48]. There have been some attempts have been made to define a “high-quality” breakfast. A study of adolescents [57] used quantitative and qualitative aspects of breakfast foods, the frequency of breakfast consumption, and the energy contribution of the breakfast meal to designate a breakfast score for defining three groups: no breakfast, or a “low quality” breakfast, or a “good/excellent quality” breakfast. Those consuming “good quality” breakfasts had higher intakes of bread, fruit, vegetables, milk, and FJ and lower intakes of soft drinks than those consuming “low quality breakfasts” [57]. Another study defined “high-quality breakfasts” as those including whole grain, fruit or FJ, and low-fat milk products or other sources of calcium [58].

In this study, children consuming breakfast patterns with significantly higher overall diet quality, when compared with breakfast skippers were generally those patterns that included grains, LFM, and fruit or FJ. In adults, it has been shown that good diet quality is essential to lowering the risk of all-cause mortality, cardiovascular disease, and cancer [59]–[61]. One would assume that the same reduction of cardiovascular disease risk would be true in children, especially adolescents; however, this has not been well studied. One study showed that scores on the dietary guideline index for children and adolescents were associated with nutrient intake, but not adiposity [62]. In a study of adolescents, a “healthy diet” coupled with physical activity has been shown to be associated with a decreased cardiovascular disease risk [63]. Finally, Papoutsou, et al. [5] showed that females, but not males, who consumed breakfast, had lower levels of cardiovascular risk factors than breakfast skippers.

Most [1], [3]–[9], [19], [21], [22]–[25], but not all [64], studies have shown that consumption of breakfast has been associated with lower weight parameters in children. Our study showed that those consuming some, but not all, of the breakfast patterns in this study had lower BMI z-scores than breakfast skippers. This is consistent with others who have made initial attempts to characterize the type of breakfast consumed with weight or adiposity parameters. Deshmukh-Taskar, et al. [3] in a study of children 9–18 years with breakfast skippers, and RTEC or “other breakfast” consumers the breakfast skippers had the highest weight, followed by those consuming “other breakfasts,” and then by RTEC consumers. Fernández Morales, et al. also showed an inverse association between BMI and consumption of calcium, fiber, dairy products, and cereal; they concluded that weight was related to “breakfast quality” [65]. Cho, et al. [66] found similar results, using NHANES III data, although their study also included adults.

Deshmukh-Taskar, et al. [3] suggested multiple reasons for the association between breakfast consumption and lower weight, including rebound overeating at other meals, diet induced thermogenesis, or consumption of more evenly distributed energy intake throughout the day. Although these reasons are plausible, the amount of energy and potentially specific foods consumed at breakfast and throughout the day clearly influence the association between breakfast consumption and weight and these associations have not been well studied.

Examining the consumption of the individual breakfast patterns with the daily total nutrient intake was of particular interest. Consumption of a “Whole Fruit” breakfast pattern gives the impression it would be inherently “healthy” and associated with an overall positive nutrient intake and weight status; however, this pattern contributed little to daily nutrient intake among consumers and had a prevalence of overweight/obese of 36%, which was not different from that seen in breakfast skippers. These findings also suggest that further studies are needed to determine associations of consumption of different breakfast patterns or breakfast skipping with gender, behavioral considerations, or socioeconomic status; the source of the breakfast meal (*i.e*. at home, at school, or at fast food restaurants); the association of specific breakfast foods/meals and subsequent intake throughout the day; and barriers to breakfast consumption in children. In this way, it may be able to determine more fully the importance of breakfast and its relationship to health.

*Study Strengths and Limitations:* NHANES is the largest available nationally representative surveillance program in the US and allows use of a very large sample size. Use of pattern analysis to determine breakfast clusters is a novel approach to the study of the breakfast meal. Limitations of this study are that NHANES data are cross-sectional; thus, cause and effect cannot be inferred. A recent meta-analysis cautioned about over-interpretation of results from cross-sectional studies that have examined breakfast consumption and weight [67]. Twenty-four hour dietary recalls have a series of inherent limitations: they may not reflect usual intake and they are memory dependent, which may lead to under-or over-reporting; however, a single 24-hour recall is sufficient to report mean group intake [68]. For children two to 11 years, proxies reported or assisted with the 24-hour recalls. Proxies, usually parents, can often report accurately what their children eat at home [69]; however, they may not know what they eat outside the home [70], which could result in reporting errors [71]. Use of standardized recipe files and food composition databases can also increase error when examining nutrient intake [72].

This study, as are all studies of breakfast, was limited by the lack of a standard definition of “breakfast” and of “breakfast skippers” [30]. In this study breakfast was self-defined; further, categorization of a child into a breakfast pattern or as a breakfast skipper was based on consumption the day of the recall only with may have led to misclassification of children into a particular breakfast pattern or as a breakfast skipper. The association of consumption of individual breakfast patterns on total daily intake may not reflect the breakfast patterns *per se*, but may be the result of what is consumed throughout the day. Lastly, there is a potential for residual confounding or to other variables not measured in the NHANES [73].

## 5. Conclusion

These data indicate that the breakfast meal has the potential to be an important meal and generally makes a positive contribution to nutrient intake, diet quality, and weight/adiposity parameters, but that care should be taken by individuals to select nutrient-dense foods, such as fruit/FJ, LFM, and fortified grain foods low in fat and added sugars. It is also important to integrate this type of nutrient-dense breakfast with an overall healthy eating plan [74]. These results, as well as the inverse association with weight and adiposity measures shown in consumers placed in some patterns, need further study to determine how breakfast meals influence energy, nutrient, and food group intakes and the timing of meals/snacks throughout the day.

## Figures and Tables

**Supplemental Table 1.** The ten most commonly consumed foods in each breakfast pattern consumed by children 2–18 years of age participating in NHANES 2001–2008.

Cluster	Cluster Name	10 Most Commonly Consumed Foods in Cluster (n=consumers)
1	Gr/LFM/Sweets/FJ	2% milk (n = 511), whole milk (n = 466), pancakes (n = 338), orange juice (n = 336), syrup blends (n = 246), waffle (n = 237), apple juice (n = 227), white toast (n = 180), jelly (n = 179), 100% maple syrup (n = 177)
2	No Breakfast	
3	PSRTEC/LFM	2% milk (n = 1136), 1% milk (n = 211), Froot Loops (n = 166), non-fat milk (n = 155), Frosted Flakes (n = 144), Honey Nut Cheerios (n = 129), Cinnamon Toast Crunch (n = 103), Lucky Charms (n = 100), Orange Juice (n = 99), Fruity Pebbles (n = 81)
4	Gr	Breakfast tart (n = 145), 2% milk (n = 58), table sugar (n = 47), waffle (n=45), white toast (n = 40), cake doughnut (n = 39), soup—mostly noodles (n = 38), sweet roll (n = 38), yeast doughnut (n = 38), pancakes (n = 34)
5	Eggs/Gr/MPF/FJ	Eggs—omelet or scrambled with fat (n = 253), fried egg (n = 196), whole milk (n = 154), eggs—omelet or scrambled with cheese (n = 142), orange juice (n = 125), white toast (n = 123), bacon (n = 116), eggs—omelet or scrambled without fat (n = 116), 2% milk (n = 112), Eggs—omelet or scrambled NS with fat (n = 76)
6	PSRTEC/Whole Milk	Whole milk (n = 1065), Froot Loops (n = 171), Frosted Flakes (n = 127), milk NFS (n = 74), Lucky Charms (n = 67), Honey Nut Cheerios (n = 65), Apple Jacks (n = 61), orange juice (n = 58), Cocoa Puffs (n = 56), Fruity Pebbles (n = 55)
7	RTEC/LFM	2% milk (n = 381), Cheerios (n = 141), 1% milk (n = 95), table sugar (n = 75), Corn flakes (n = 75), Rice Krispies (n = 66), banana (n = 52), non-fat milk (n = 52), Kix (n = 40), orange juice (n = 33)
8	Soft Drink/FJ/Gr/Potatoes	2% milk (85), orange juice (n = 83), hash browns (n = 63), cola (n = 60), fruit juice drink (n = 59), low-fat fruit yogurt (n = 51), ketchup (n = 51), whole milk (n = 48), orange juice (n = 47), vitamin fortified fruit drink (n = 47)
9	RTEC/Whole Milk	Whole milk (n = 656), Cheerios (n = 112), corn flakes [Kellogg's] (n = 110), table sugar (n = 97), banana (n = 50), milk NFS (n = 48), corn flakes NFS (n = 40), Rice Krispies (n = 39), Kix (n = 38), orange juice (n = 28)
10	MPF/Gr/FJ	Ketchup (n = 75), whole milk (n = 69), bacon (n = 58), pork sausage (n = 53), orange juice (n = 49), white bread (n = 46), sausage on biscuit (n = 41), apple juice (n = 39), jelly (n = 37), pancakes (n = 36)
11	Cooked Cereal/Milk/FJ	Instant oatmeal no fat added in cooking (n = 181), sugar (n = 79), 2% milk (n = 60), whole milk (n = 57), regular oatmeal no fat added in cooking (n = 48), quick cooking oatmeal no fat added in cooking (n = 37), orange juice (n = 20), instant oatmeal fat added in cooking (n = 19), brown sugar (n = 19), orange juice with calcium (n = 17)
12	Whole Fruit	Apple (n = 39), banana (n = 36), orange (n = 12), NS grapes (n = 8), strawberries (n = 6), mango (n = 3), tangerine (n = 3), NS applesauce (n = 3), table sugar (n = 3), pear (n = 2)

Abbreviations: Gr = Grain; LFM = lower fat milk; FJ = fruit juice; PSRTEC = presweetened ready-to-eat cereal; RTEC = ready-to-eat cereal; MPF = meat, poultry, fish; NS = not specified; NFS = not further specified. In 10 most commonly consumed foods, all milk refers to cow's fluid milk; all eggs are chicken eggs; bacon is pork bacon.

**Supplemental Table 2.** The ten foods in each breakfast pattern consumed by children 2–18 years of age participating in NHANES 2001-2008 that contributed the most energy.

Cluster	Cluster Name	10 Food Contributing the Most Weighted Energy (kcals)
1	Gr/LFM/Sweets/FJ	Pancakes (68,247), Whole Milk (68,055), 2% milk (65,496), Waffle (56,182), OJ (36, 242), Syrup Blends (34,709), Breakfast Tart (34,254), French Toast (30,000), Maple Syrup (25,471), Biscuit (24,852)
2	No Breakfast	
3	PSRTEC/LFM	2% Milk (134,720), Frosted Flakes (27,203), Cinnamon Toast Crunch (22,805), Froot Loops (22,113), 1% Milk (20,276), Honey Nut Cheerios (19,503), Fruity Pebbles (15,928), Lucky Charms (13,913), Cocoa Puffs (12,293), Skim Milk (11,683)
4	Gr	Breakfast Tart (50,919), Cake Doughnut (15,059), Cinnamon Bun (13,880), Yeast Doughnut (13,384), Waffle (12,238), Muffin (11,650), Thin Crust Meat Pizza (10,065), Pancakes (9,998), Thick Crust Meat Pizza (9,547), Soup, Mostly Noodles (9349)
5	Eggs/Gr/MPF/FJ	Omelet or Scrambled Egg, fat added (46,380); Cheese Omelet or Scrambled Egg (34,025); Fried Egg (29,778); Whole Milk (22,771); Omelet or Scrambled Egg, no fat added (20,029); Ham or Bacon Omelet or Scrambled Egg (16,523); Omelet or Scrambled Egg, NS fat added (15,302); OJ (14,324); 2% Milk (13,812); Pancakes (12,276)
6	PSRTEC/Whole Milk	Whole Milk (142,172), Froot Loops (29,114), Frosted Flakes Kellogg's (26,187), Cinnamon Toast Crunch (12,710), Frosted Flakes NFS (10,624), Apple Jacks (10,358), Honey Nut Cheerios (10,304), Lucky Charms (9,546), Fruity Pebbles (9,525), Cocoa Puffs (9,037)
7	RTEC/Low-Fat Milk	2% Milk (49,209), Cheerios (17,807), Cocoa (9,932), 1% Milk (9,355), Corn Flakes (9,108), Rice Krispies (8,524), Reduced fat milk Cocoa (6,604), Banana (4,435), Skim Milk (3,757), Whole Chocolate Milk (3,698)
8	Soft Drink/FJ/Gr/Potatoes	Hash Browns (16,008), Home Fries (11,468), Cola (10,831), OJ (10,811), 2% Milk (9,317), Fruit Drink (8,606), Low fat fruit yogurt (8,606), Vitamin Fortified Fruit Drink (7,529), Fruit Soft Drink (6,617), Calcium Fortified OJ (6,558)
9	RTEC/Whole Milk	Whole Milk (105,861), Cheerios (14,796), Corn Flakes Kellogg's (14,337), Milk NFS (6,687), Rice Krispies (4,921), Corn Flakes NFS (4,622), Banana (4,563), Table Sugar (3,200), Kix (3,198), Apple Juice (2,668)
10	MPF/Gr/FJ	Sausage Biscuit (17,892), Pork Sausage (13,331), Whole Milk (10,804), French Fries (9,585), Bacon (8,308), Sausage & Cheese on English Muffin (7,453), Sausage (not cold cut) (6,105), White Bread (5,804), OJ (5,646), Corn Dog (5,382)
11	Cooked Cereal/Milk/FJ	Instant Oatmeal no Fat Added in Cooking (42,943), Regular Oatmeal no Fat Added in Cooking (9,856), Quick Cooking Oatmeal no Fat Added in Cooking (7,706), Whole Milk (6,467), Instant Oatmeal Fat Added in Cooking (5,963), 2% Fat (5,927), Table Sugar (3,800), OJ (2,492), Regular Oatmeal Fat Added in Cooking (2,324), Whole Wheat Cereal no Fat Added in Cooking (2,160)
12	Whole Fruit	Banana (3,777), Apple (2,983), Orange (889), Grapes NS (577), Mango (330), Applesauce (303), Baby Food—Peaches (221), Cantaloupe (212), Fruit Cocktail Drained Solids (197), Fruit Cocktail in Light Syrup (193)

Abbreviations: Gr = Grain; LFM = lower fat milk; FJ = fruit juice; PSRTEC = presweetened ready-to-eat cereal; RTEC = ready-to-eat cereal; MPF = meat, poultry, fish; NS = not specified; NFS = not further specified. In 10 most commonly consumed foods, all milk refers to cow's fluid milk; all eggs are chicken eggs; bacon is pork bacon.
